# Radiofrequenzdenervation der Wirbelsäule und des Iliosakralgelenks: Eine systematische Literaturrecherche nach GRADE mit neuer S3-Leitlinie

**DOI:** 10.1007/s00482-024-00799-w

**Published:** 2024-03-01

**Authors:** Stephan Klessinger, Hans-Raimund Casser, Sebastian Gillner, Holger Koepp, Andreas Kopf, Martin Legat, Katharina Meiler, Heike Norda, Markus Schneider, Matti Scholz, Phillipp J. Slotty, Volker Tronnier, Martin Vazan, Karsten Wiechert

**Affiliations:** 1 Neurochirurgie Biberach, Eichendorffweg 5, 88400 Biberach, Deutschland; 2 Klinik für Neurochirurgie, Universitätsklinikum Ulm, Albert-Einstein-Allee 23, 89081 Ulm, Deutschland; 3 DRK Schmerz-Zentrum, Auf der Steig 14, 55131 Mainz, Deutschland; 4 Klinik für Anästhesiologie, Intensiv- und Notfallmedizin, Sana Krankenhaus Benrath, Gräulicher Str. 120, 40625 Düsseldorf, Deutschland; 5 Wirbelsäulenzentrum, St. Josefs-Hospital, Beethovenstr. 20, 65189 Wiesbaden, Deutschland; 6 Klinik für Anästhesiologie mit Schwerpunkt operative Intensivmedizin, Campus Benjamin Franklin, Charité-Universitätsmedizin Berlin, Hindenburgdamm 30, 12200 Berlin, Deutschland; 7 Schmerzzentrum Zofingen, Hintere Hauptgasse 9, 4800 Zofingen, Schweiz; 8 Orthopädie, Städtisches Klinikum Magdeburg, Birkenallee 34, 39130 Magdeburg, Deutschland; 9 UVSD SchmerzLOS e. V., Fürsthof 24, 24534 Neumünster, Deutschland; 10 alphamed Bamberg, Kärntenstr. 2, 96052 Bamberg, Deutschland; 11 ATOS Orthopädische Klinik Braunfels GmbH & Co. KG, Hasselbornring 5, 35619 Braunfels, Deutschland; 12 Klinik für Neurochirurgie, Universitätsklinikum Düsseldorf, Moorenstr. 5, 40225 Düsseldorf, Deutschland; 13 Klinik für Neurochirurgie, Universitätsklinikum Schleswig-Holstein, Ratzeburger Allee 160, 23562 Lübeck, Deutschland; 14 Praxis für Neurochirurgie, Wirbelsäulen- und Rückenzentrum Dresden, Borsbergstr. 44, 01309 Dresden, Deutschland; 15 Rückenzentrum am Michel, Ludwig-Erhard-Str. 18, 20459 Hamburg, Deutschland

**Keywords:** Perkutane Neurotomie, „Medial branch block“, Spezifische Rückenschmerzen, Nackenschmerzen, Wirbelsäule, Percutaneous neurotomy, Medial branch block, Specific back pain, Neck pain, Spine

## Abstract

**Hintergrund:**

In diesem Artikel werden die Ergebnisse der S3-Leitlinie „Radiofrequenzdenervation der Facettengelenke und des ISG“ zusammengefasst. Die vorhandene Evidenz zur Indikation, zu Testblockaden und zu technischen Parametern wird dargelegt.

**Ziel:**

Es soll sowohl einer Über- als auch eine Unterversorgung entgegengewirkt werden, was auch sozioökonomische Bedeutung hat.

**Material und Methode:**

Es erfolgte eine systematische Auswertung der Literatur nach den Grading of Recommendations, Assessment, Development, and Evaluation (GRADE). Eine multidisziplinäre Leitliniengruppe hat Empfehlungen und Statements ausgearbeitet.

**Ergebnisse:**

Für 20 Schlüsselfragen wurden Statements und Empfehlungen formuliert. Es gab 87,5 % Konsens für eine Empfehlung und 100 % Konsens für alle weiteren Empfehlungen und Statements. Die Leitlinie wurde von allen beteiligten Fachgesellschaften konsentiert. Die spezifischen Fragen beinhalten den Wert von Anamnese, Untersuchung und Bildgebung, die Notwendigkeit einer konservativen Therapie vor einer Intervention, die Bedeutung von Testblockaden („medial branch block“ und „lateral branch block“), die Wahl der Bildgebung für eine Denervation, die Wahl der Trajektorie, die Möglichkeit, die Größe der Läsion zu beeinflussen, Stimulation, die Möglichkeit einer Wiederholung, Sedierung und Entscheidungshilfen zu Patienten mit Antikoagulanzien, Metallimplantaten und Schrittmachern und Hinweise zur Vermeidung von Komplikationen.

**Schlussfolgerungen:**

Ausgewählte Patienten können von einer gut durchgeführten Radiofrequenzdenervation profitieren. Die Empfehlungen der Leitlinie basieren auf sehr niedriger bis moderater Qualität der Evidenz.

## Abstract (en)

**Background:**

This article summarizes the results of the German guideline on radiofrequency denervation of the facet joints and the sacroiliac joint. Evidence on the indications, test blocks and technical parameters are presented.

**Objective:**

The aim is to avoid overtreatment and undertreatment, which is also of socioeconomic importance.

**Material and method:**

A systematic evaluation of the literature was carried out according to the grading of recommendations assessment, development and evaluation (GRADE) approach. A multidisciplinary guideline group has developed recommendations and statements.

**Results:**

Statements and recommendations were given for 20 key questions. There was an 87.5% consensus for 1 recommendation and 100% consensus for all other recommendations and statements. The guideline was approved by all scientific medical societies involved. Specific questions included the value of the medical history, examination and imaging, the need for conservative treatment prior to an intervention, the importance of test blocks (medial branch block and lateral branch block), choice of imaging for denervation, choice of trajectory, the possibility to influence the size of the lesion, stimulation, the possibility of revision, sedation and decision support for patients with anticoagulants, metal implants and pacemakers and advice on how to avoid complications.

**Conclusion:**

Selected patients can benefit from well-performed radiofrequency denervation. The guideline recommendations are based on very low to moderate quality of evidence.

Die Ursachen für chronischen Rückenschmerz werden kontrovers diskutiert und es ist üblich, zwischen spezifischen und nichtspezifischen Rückenschmerzen zu unterscheiden [3, 5], Die Leitlinie „Spezifischer Kreuzschmerz“ [5] nennt die Facettengelenke und das Iliosakralgelenk als mögliche spezifische Schmerzursache und sie empfiehlt, eine Radiofrequenzdenervation bei Patienten mit einem Facettensyndrom in Erwägung zu ziehen. Allerdings werden keine Hinweise gegeben, wie die Diagnose Facettensyndrom gesichert und die Denervation durchgeführt werden sollte.

Untersucht man die wenigen randomisiert-kontrollierten Studien (RCT), die die Ergebnisse einer Radiofrequenzdenervation (RF-Denervation) mit einer Sham-Prozedur verglichen haben, so finden sich sehr heterogene Ergebnisse. Bei den vier RCT, die eine RF-Denervation an der Halswirbelsäule untersucht haben, ist die Qualität der Evidenz moderat bis sehr niedrig [13, 18, 20, 25]. Die Patientenzahlen sind gering und es wurden unterschiedliche Patientengruppen untersucht (Verkehrsunfall, Nackenschmerzen, Kopfschmerzen). In einer Studie konnte jedoch ein deutlicher Vorteil nach RF-Denervation bezüglich Schmerzfreiheit herausgearbeitet werden. Die aktuellste Studie fand jedoch keinen Effekt.

Acht RCT haben die RF-Denervation an der Lendenwirbelsäule untersucht [7, 10, 12, 16, 19, 21, 23, 24], mit sehr niedriger bis moderater Qualität der Evidenz. Bessere Ergebnisse im Vergleich zur Sham-Gruppe finden sich vor allem bis zu 3 Monate nach der Intervention. Die Ergebnisse sind allerdings heterogen, da unterschiedliche Indikationen und unterschiedliche Techniken der Denervation verwendet wurden.

Am Iliosakralgelenk (ISG) finden sich zwar bessere Ergebnisse nach Denervation im Vergleich zur Sham-Gruppe nach mehr als sechs Monaten, die vier RCT [4, 15, 17, 22] unterscheiden sich aber erheblich in den Indikationen und den verwendeten Techniken der Denervation.

Aufgrund der heterogenen Ergebnisse der Studien ist es schwierig, die Wirksamkeit einer RF-Denervation einzuschätzen. Zudem geht aus den Studien nicht klar hervor, welche Indikationen für eine RF-Denervation geeignet sind und wie die RF-Denervation durchgeführt werden sollte. Daher hat die Deutsche Wirbelsäulengesellschaft die Erstellung dieser Leitlinie in Auftrag gegeben, um die vorhandene Evidenz systematisch auszuwerten und Empfehlungen zur Indikation und Durchführung einer RF-Denervation zu geben.

Die Indikation zur RF-Denervation besteht bei einem spezifischen, chronischen, therapieresistenten Schmerz, dessen Ursprung in den Facettengelenken oder dem ISG liegt. Zur Sicherung der Diagnose werden neben Anamnese, Untersuchung und Bildgebung in der Regel Testblockaden durchgeführt. Allerdings werden in den vorhandenen Studien sowohl intraartikuläre Blocks als auch „medial branch blocks“ verwendet. Der Schwellenwert der Schmerzreduktion, damit ein Block als positiv gewertet werden kann, schwankt zwischen 50 und 100 %. In manchen Studien wurde nur ein Testblock durchgeführt, in anderen mehrere oder vergleichende Blocks mit unterschiedlich lang wirksamen Lokalanästhetika.

Ziel der RF-Denervation ist es, die Schmerzweiterleitung aus dem Gelenk zu unterbrechen. Unterschiedliche Arten von Sonden an unterschiedlichen Positionen mit verschiedenen technischen Parametern (Temperatur, Dauer der Anwendung, Durchmesser der Kanüle) wurden in den Studien verwendet.

Die S3-Leitlinie zur Radiofrequenzdenervation gibt klare, evidenzbasierte Empfehlungen sowohl zur Indikation als auch zur Durchführung. Zudem wird auf Antikoagulanzien, vorhandene Metallimplantate oder implantierte elektrische Geräte eingegangen.

## Methodik der Leitlinienentwicklung

Die Leitlinie wurde von der Deutschen Wirbelsäulengesellschaft (DWG) beauftragt. Es wurde eine repräsentative Leitliniengruppe (Infobox 1) aus Fachärzten für Neurochirurgie, Orthopädie und Unfallchirurgie und Anästhesie gebildet, zudem wurde eine Patientenvertreterin beteiligt.

Die Methodik der Leitlinie richtet sich nach dem AWMF-Regelwerk. Die Beurteilung der Evidenz erfolgte nach dem GRADE-System (Grading of Recommendations, Assessment, Development and Evaluation; [9]). Für die 20 Schlüsselfragen der Leitlinie wurde die PICO-Struktur („patients, intervention, control, outcome“) berücksichtigt (Infobox 2; [8]).

Bei der systematischen Literatursuche konnte ein Cochrane Review aus dem Jahr 2015 [14] ausgewertet werden, zudem wurden vorhandene Leitlinien in die Literatursuche einbezogen und zusätzlich erfolgte eine Literatursuche über Medline und Cochrane.

Die Empfehlungen und Statements der Leitlinie wurden in einem interdisziplinären Konsensverfahren unter neutraler Moderation abgestimmt. Für eine Empfehlung gab es 87,5 % Konsens, für alle weiteren 100 %. Die endgültige Fassung der Leitlinie wurde nach der AGREE-II-Systematik (Appraisal of Guidelines for Research & Evaluation II; [2]) beurteilt.

Für alle Schlüsselfragen werden Statements oder Empfehlungen genannt, wobei mit A eine starke Empfehlung (soll), mit B eine schwache Empfehlung (sollte) und mit 0 eine offene Empfehlung (kann erwogen werden) bezeichnet wird. Existiert keine Evidenz, so wurde ein Expertenkonsens (EK) vermerkt [1].

Details zur Methodik, zu den PICO-Fragen (Infobox 2) und zur Literatursuche finden sich im Leitlinienreport [11].

## Statements und Empfehlungen der Leitlinie

Die Statements und Empfehlungen der Leitlinie beziehen sich auf die Indikation zur RF-Denervation und auf die konkrete Durchführung, da für diese beiden Gebiete die Literatur keine einheitlichen Vorgaben liefert. Es ist davon auszugehen, dass eine strenge Indikation bessere Ergebnisse liefert, allerdings würde bei einer zu strengen Indikation Patienten mit einem spezifischen Rückenschmerz eine potenziell wirksame Therapie vorenthalten. Bei der RF-Denervation ist davon auszugehen, dass eine größere Läsion eine längere Strecke des Nerven verödet, wodurch eine längere Wirkdauer zu erwarten ist. Daher sollte die Durchführung der RF-Denervation diesbezüglich optimiert werden.

Die ausführlichen Ergebnisse mit allen Angaben zur verwendeten Literatur finden sich in der Leitlinie und in den Evidenztabellen zur Leitlinie [11].

### Anamnese, Untersuchung und Bildgebung

Die Diagnose eines Facettengelenkschmerzes oder eines ISG-Schmerzes wird mittels Anamnese, Untersuchung, Bildgebung und Testblockaden gestellt. Da Testblockaden aufwendig und invasiv sind und somit ein Risiko für den Patienten darstellen und zudem auch falsch-positive und falsch-negative Ergebnisse zu erwarten sind, ist es wichtig, die idealen Patienten für Testblockaden herauszufinden.

#### Statements und Empfehlungen

- Es gibt keine Symptome oder klinischen Untersuchungen, die für das Vorliegen eines zervikalen oder lumbalen Facettengelenkschmerzes pathognomonisch sind.
- Schmerzkarten und die Ergebnisse der segmentalen klinischen Untersuchung sollten zur Identifizierung der Level zur Austestung herangezogen werden (Empfehlungsgrad: B).
- Die Evidenz ist widersprüchlich, ob die Kombination mehrerer klinischer Provokationstests (≥ 3 Tests) in der Lage ist, das Ergebnis einer intraartikulären ISG-Injektion vorherzusagen. Beim Fehlen von positiven Provokationstests sollte keine Testblockade am ISG durchgeführt werden (Empfehlungsgrad: B).
- Es gibt keine ausreichende Evidenz dafür, symptomatische Facettengelenke oder ein symptomatisches ISG allein mittels bildgebender Verfahren zu diagnostizieren.
- Bildgebende diagnostische Verfahren haben keinen prädiktiven Wert bezüglich der Ergebnisse von diagnostischen Blockaden oder des Ergebnisses einer RF-Denervation. Sie liefern aber relevante Informationen zu differenzialdiagnostischen Erkrankungen.
- Vorhandene Bildgebung sollte neben der Anamnese und dem klinischen Untersuchungsbefund bei der Entscheidung, welche Level behandelt werden, berücksichtigt werden (Empfehlungsgrad: B).

### Diagnostische Blockaden

Die Austestung vor einer RF-Denervation sollte nur bei Patienten mit einer chronischen therapieresistenten Schmerzsymptomatik erfolgen. Eine chronische Schmerzstörung mit somatischen und psychischen Faktoren muss berücksichtigt werden.

Unterschiedliche Zielpunkte werden für diagnostische Blockaden an der Hals- und Lendenwirbelsäule verwendet. In Frage kommen sowohl die Nervenäste, die das Gelenk versorgen („medial branch“), es werden aber auch Testblockaden in die Gelenkkapsel oder in den Gelenkspalt durchgeführt. Es ist zu überlegen, ob die Testblockade und die RF-Denervation an der gleichen anatomischen Struktur („medial branch“) durchzuführen sind. Für das ISG wird diskutiert, ob eine intraartikuläre Injektion oder ein „lateral branch block“ sinnvoll ist.

Zur Testblockade werden verschiedene Lokalanästhetika mit unterschiedlich langer Wirkdauer teils als vergleichende Blocks verwendet. Wichtig ist es, das verwendete Volumen klein zu halten, damit der Block spezifisch ist. Kontrastmittel zeigt die zu erwartende Verteilung des Medikaments.

Bei der Auswertung der Testblockade werden unterschiedliche Schwellenwerte zwischen 50 und 100 % als positiv gewertet werden. Eine Schmerzreduktion von 50 % wird bereits als substanzielle Verbesserung angesehen [6] und ermöglicht mehr Patienten den Zugang zur Therapie. Bessere Ergebnisse einer RF-Denervation könnten bei strengerer Indikation erwartet werden.

Die Zahl falsch-positiver Tests lässt sich durch eine Wiederholung der Testblockade reduzieren.

#### Statements und Empfehlungen

- Vor einer Austestung der Facettengelenke und einer RF-Denervation soll eine chronische Schmerzsymptomatik bestehen (Empfehlungsgrad: A). Bei Patienten mit Verdacht auf eine chronische Schmerzstörung mit somatischen und psychischen Faktoren bedarf es vor einer Intervention einer interdisziplinären Abklärung (Empfehlungsgrad: EK).
- „Medial branch blocks“ sind als Testblockade vor einer RF-Denervation geeignet. Zur Testblockade vor einer RF-Denervation sollten „medial branch blocks“ durchgeführt werden (Empfehlungsgrad: B).
- Als primäre (therapeutische) Intervention bei ISG-Schmerzen kann eine intraartikuläre Injektion durchgeführt werden (Empfehlungsgrad: 0).
- Zur Austestung vor einer RF-Denervation sollten „multi-site, multi-depth lateral branch blocks“ durchgeführt werden (Empfehlungsgrad: B). Es kann eine intraartikuläre Injektion zur Austestung dienen (Empfehlungsgrad: 0).
- Für eine diagnostische Testblockade kann sowohl ein kurz als auch ein lang wirksames Lokalanästhetikum verwendet werden (Empfehlungsgrad: 0). Das verwendete Volumen sollte weniger als 0,5 ml betragen (Empfehlungsgrad: B).
- Für einen diagnostischen „medial branch block“ sollte Kontrastmittel verwendet werden (Empfehlungsgrad: B).
- Ein Schwellenwert von 50 % Schmerzreduktion nach einem „medial branch block“ sollte verwendet werden, damit der Testblock als positiv gewertet werden kann (Empfehlungsgrad: B).
- Vor einer RF-Denervation sollten in der Regel zwei diagnostische „medial branch blocks“ mit positivem Ansprechen durchgeführt werden (Empfehlungsgrad: B).

### Durchführung der RF-Denervation

Die Position der Elektrode muss bei einer RF-Denervation mittels Bildgebung kontrolliert werden. Grundsätzlich kommen Durchleuchtung, CT und Ultraschall infrage. Eine große Läsion wird angestrebt, um einen möglichst langen Anteil des Nerven zu veröden, allerdings sollte die Läsion nicht so groß sein, dass unbeabsichtigt Nachbarstrukturen verletzt werden. Durch Wahl der Temperatur, der Dauer der Wärmeeinwirkung, des Durchmessers der Kanüle und der Länge der aktiven Spitze kann die Läsionsgröße beeinflusst werden. Es wurde versucht, die Größe der Läsion durch Veränderungen der Elektrodenspitze zu verbessern, zudem kommen auch bipolare Techniken infrage. Relevant für die Größe der Läsion kann auch der Winkel der Elektrode zum Nerven sein (eher rechtwinklige oder eher parallele Nadellage). Die Positionierung der Elektrode kann durch eine sensorische oder motorische Teststimulation zusätzlich zur anatomischen Kontrolle in der Bildgebung kontrolliert werden.

Generell ist zu überlegen, ob eine Wiederholung der RF-Denervation anzubieten ist.

Bei Patienten mit Antikoagulanzien oder Thrombozytenaggregationshemmern muss das Risiko bei Fortführen der Medikation mit dem Risiko bei Absetzen abgewogen werden. Bei ausgewählten Patienten kann eine Sedierung erwogen werden.

Besondere Überlegungen sind bei Patienten mit Metallimplantaten (z. B. Spondylodese) notwendig und bei Patienten mit medizinischen Implantaten, da das elektrische Feld zu Störungen führen kann.

#### Statements und Empfehlungen

- Eine RF-Denervation sollte mit Durchleuchtung durchgeführt werden (Empfehlungsgrad: B). Die diagnostische Testblockade sollte mit der gleichen Bildgebung durchgeführt werden wie später die RF-Denervation (Empfehlungsgrad: B).
- Es ist davon auszugehen, dass eine größere Läsion zu einer länger anhaltenden Schmerzreduktion führt.
- Bei konventionellen Sonden sollten eine Temperatur von 75 bis 90 °C (Empfehlungsgrad: B) und eine Dauer von 60 bis 120 s (Empfehlungsgrad: EK) sowie ein Durchmesser der Kanüle von mindestens 18 G (Empfehlungsgrad: EK) mit einer aktiven Spitze von 10 mm (oder auch 5 mm an der Halswirbelsäule) für die Denervation verwendet werden (Empfehlungsgrad: EK). Bei der Verwendung von konventionellen Sonden werden mehrere Läsionen pro „medial branch“ bzw. „lateral branch“ empfohlen (Empfehlungsgrad: EK).
- Eine Empfehlung für oder gegen die Verwendung eines bestimmten Sondentyps kann nicht gegeben werden.
- An der Halswirbelsäule soll bei Verwendung einer konventionellen Elektrode eine parallele Nadellage über einen posterioren bzw. schräg-posterioren Zugang ggf. mit einer gebogenen Nadel gewählt werden (Empfehlungsgrad: A). An der Lendenwirbelsäule sollte bei Verwendung einer konventionellen Elektrode eine parallele Nadellage gewählt werden (Empfehlungsgrad: B).
- Eine sensorische und motorische Stimulation kann durchgeführt werden (Empfehlungsgrad: 0).
- Nach einer erfolgreichen RF-Denervation mit mindestens 50 % Schmerzreduktion für mindestens 3 Monate sollte bei Wiederauftreten der gleichen Schmerzen eine erneute RF-Denervation angeboten werden (Empfehlungsgrad: B). War eine technisch korrekte RF-Denervation nicht erfolgreich, sollte keine erneute RF-Denervation der gleichen „medial branches“ durchgeführt werden (Empfehlungsgrad: B). Bei Wiederauftreten der gleichen Schmerzen nach erster erfolgreicher RF-Denervation sollte vor einer erneuten RF-Denervation keine erneute Austestung mittels „medial branch block“ erfolgen (Empfehlungsgrad: B).
- Es besteht keine Evidenz dafür, dass das Fortführen der Medikation mit Antikoagulanzien oder Thrombozytenaggregationshemmern zu einem klinisch relevanten erhöhten Blutungsrisiko führt. Vor einer RF-Denervation an der Lendenwirbelsäule oder am ISG sollten Antikoagulanzien oder Thrombozytenaggregationshemmer nicht abgesetzt werden, wobei immer eine Einzelfallentscheidung bezüglich des Risikos und des Nutzens erfolgen muss (Empfehlungsgrad: B). Bei einer RF-Denervation an der Halswirbelsäule sollte eine individuelle Risiko/Nutzen-Abwägung für jeden einzelnen Patienten getroffen werden (Empfehlungsgrad: B).
- Eine Sedierung, bei der der Patient ansprechbar ist, kann bei einer RF-Denervation durchgeführt werden (Empfehlungsgrad: 0).
- Bei Patienten mit Metallimplantaten sollte eine multiplanare fluoroskopische Darstellung genutzt werden zur Darstellung des Implantats und der Sonde, wobei ein direkter Kontakt der Sonde mit dem Metall vermieden werden sollte (Empfehlungsgrad: B).
- Eine RF-Denervation bei Patienten mit implantiertem Schrittmacher oder Defibrillator soll in enger Zusammenarbeit mit einem Kardiologen erfolgen (Empfehlungsgrad: A). Die Neutralelektrode sollte so platziert werden, dass das elektrische Feld zwischen Läsionselektrode und Neutralelektrode den Schrittmacher/Defibrillator nicht tangiert (Empfehlungsgrad: B). Für die Dauer einer RF-Denervation sollte ein implantierter Neurostimulator ausgeschaltet werden (Empfehlungsgrad: EK).
- Schwerwiegende Komplikationen sind nach RF-Denervationen sehr selten. Es ist wichtig, bei einer RF-Denervation an der Halswirbelsäule die vorhandene Bildgebung bezüglich anatomischer Varianten in die Planung einzubeziehen (Empfehlungsgrad: EK). Es ist wichtig, die Patienten vor einer RF-Denervation über die Möglichkeit einer vorübergehenden Schmerzzunahme oder länger anhaltender Schmerzen und Dysästhesien zu informieren. Schmerzen, die länger als zwei Wochen anhalten, sind sehr selten (Empfehlungsgrad: EK).

## Diskussion

Die vorhandenen klinischen Studien zur RF-Denervation zeigen sehr heterogene Ergebnisse, was vor allem an der unterschiedlichen Auswahl geeigneter Patienten und an unterschiedlicher verwendeter Technik liegt. Daher wurde diese multidisziplinäre Leitlinie entwickelt, um die Ergebnisse einer RF-Denervation zu verbessern aber auch um unnötige Testblockaden und Denervationen zu vermeiden.

Zu den Stärken dieser Leitlinie gehört die strikte Einhaltung des GRADE-Ansatzes. Die Leitliniengruppe bestand aus einer repräsentativen Auswahl von Klinikern und Wissenschaftlern und wurde von der AWMF einem Review nach der AGREE-Systematik unterzogen. Für nahezu alle Empfehlungen und Statements gab es 100 % Konsens.

Limitierungen bestehen durch teilweise nur niedrige Qualität der Evidenz in der Literatur. Die Auswahl der 20 Schlüsselfragen erhebt keinen Anspruch auf Vollständigkeit. Zu manchen Fragen gibt es kontroverse Diskussionsmöglichkeiten.

### Infobox 1 Beteiligte Fachgesellschaften und deren Vertreter

- Herausgebende Fachgesellschaft
  - Deutsche Wirbelsäulengesellschaft (DWG): Stephan Klessinger (Koordinator der Leitlinie), Karsten Wiechert (Mandatsträger)
- Beteiligte Fachgesellschaften (Mandatsträger)
  - Deutsche Gesellschaft für Anästhesiologie und Intensivmedizin e. V. (DGAI): Andreas Kopf
  - Deutsche Gesellschaft für Neurochirurgie (DGNC): Volker Tronnier
  - Deutsche Gesellschaft für Neuromodulation (DGNM): Phillipp Slotty
  - Deutsche Gesellschaft für Orthopädie und Unfallchirurgie e. V. (DGOU): Matti Scholz
  - Deutsche Schmerzgesellschaft e. V.: Hans-Raimund Casser
  - Interdisziplinäre Gesellschaft für orthopädische/unfallchirurgische und allgemeine Schmerztherapie e. V. (IGOST): Markus Schneider
- Patientenvertretung
  - Unabhängige Vereinigung aktiver Schmerzpatienten in Deutschland e. V. (SchmerzLOS): Heike Norda

### Infobox 2 Die 20 Schlüsselfragen der Leitlinie nach dem PICO-Prinzip

- Anamnese/Untersuchung
  1. Kann ein ISG- oder Facettengelenkschmerz durch Anamnese und klinische Untersuchung diagnostiziert werden?
- Bildgebung
  2. Gibt es eine Korrelation zwischen Befunden der Bildgebung und dem Ergebnis einer RF-Denervation?
- Diagnostische Blockaden
  3. Ist es notwendig, vor diagnostischen Blocks konservative Therapie durchgeführt zu haben, wie lange?
  4. Sind „medial branch blocks“ zur Diagnose eines Facettengelenkschmerzes geeignet?
  5. Sind „medial blocks“ besser als intraartikuläre/perikapsuläre Blocks zur Diagnose eines Facettengelenkschmerzes geeignet?
  6. Welche diagnostischen Blocks sollten am ISG vor einer Denervation erfolgen?
  7. Welche Medikamente sind für eine Testblockade sinnvoll?
  8. Welche Schmerzreduktion nach einem therapeutischen Block wird als positiv gewertet?
  9. Wie viele diagnostische Blocks sind notwendig vor einer Denervation?
- Durchführung der RF-Denervation
  10. Welche Bildgebung wir für die RF-Denervation empfohlen?
  11. Ist die Größe der Läsion entscheidend für das Ergebnis der Denervation? Wie kann die Läsion vergrößert werden?
  12. Welche Art der Sonde ist vorteilhaft?
  13. Ist die Lage der Sonde zum Nerven (parallel, rechtwinklig) entscheidend?
  14. Ist eine sensorische oder motorische Teststimulation notwendig?
  15. Kann eine RF-Denervation wiederholt werden, nach welcher Zeit?
  16. Müssen Antikoagulanzien und Thrombozytenaggregationshemmer vor einer RF-Denervation abgesetzt werden?
  17. Ist eine Sedierung bei einer RF-Denervation sinnvoll?
  18. Ist eine RF-Denervation bei Patienten mit Metallimplantaten möglich?
  19. Ist eine RF-Denervation bei Patienten mit Herzschrittmacher oder SCS möglich?
  20. Was sind typische Komplikationen einer RF-Denervation?

## Fazit für die Praxis

- Die S3-Leitlinie „Radiofrequenzdenervation der Facettengelenke und des ISG“ wurde nach systematischer Literaturauswertung und Metaanalyse nach GRADE erstellt.
- Eine repräsentative, multidisziplinäre Leitliniengruppe hat zu 20 Schlüsselfragen Empfehlungen und Statements ausgearbeitet.
- Für eine Empfehlung gab es 87,5 % Konsens, für alle anderen 100 %.
- Wichtige Fragen zur Indikation und Patientenauswahl sowie zur Durchführung einer RF-Denervation werden angesprochen.
- Neben der Lang- und Kurzfassung wurde auch eine Leitlinie für Patienten erstellt.
- Die Leitlinie gibt konkrete Empfehlungen zur Diagnostik und Durchführung einer RF-Denervation und kann daher als eine Anleitung zum evidenzbasierten Arbeiten genutzt werden.
